# Chikungunya Fever, Mauritius, 2006

**DOI:** 10.3201/eid1402.071024

**Published:** 2008-02

**Authors:** Sanjay Beesoon, Ellen Funkhouser, Navaratnam Kotea, Andrew Spielman, Rebecca M. Robich

**Affiliations:** * University of Mauritius, Reduit, Mauritius; † University of Alabama at Birmingham School of Medicine, Birmingham, Alabama, USA; ‡ Harvard School of Public Health, Boston, Massachusetts, USA; 1 Deceased.

**Keywords:** Chikungunya fever, Aedes albopictus, Mauritius Island, Rodrigues Island, deaths, letter

**To the Editor:** When an outbreak of chikungunya fever swept across several Indian Ocean islands in 2005 and 2006, the international public health community’s attention was drawn to chikungunya virus (genus *Alphavirus*, family *Togaviridae*). Among those areas affected was the Republic of Mauritius, located ≈900 km east of Madagascar, which has an estimated population of 1,250,000 and comprises the main island of Mauritius (area ≈1,865 km^2^) and several outlying islands including Rodrigues (≈180 km^2^). Mauritius Island had an initial outbreak of ≈3,500 suspected cases of chikungunya fever from April through June 2005 (*1*). With the onset of the drier season of winter, transmission of the virus subsided, but increased again during 2006. An outbreak began on Rodrigues Island in February 2006. The intensity of chikungunya fever outbreaks on Mauritius and Rodrigues Islands led us to explore the extent to which these outbreaks might have contributed to overall death rates.

Chikungunya virus is transmitted to humans primarily by the bite of infected *Aedes* spp. mosquitoes. *Aedes aegypti* was effectively eliminated from the island of Mauritius during a malaria control campaign from 1949 through 1951 (*2*). However, *A*. *albopictus* is widely distributed on the island in rural and urban habitats (*3*); thus, *A*. *albopictus* may be the most likely vector for chikungunya virus. Common peridomestic breeding places in Mauritius include small pots and vases (used to decorate the outside of homes) and discarded rubber tires (*3*).

Chikungunya fever was first recognized in 1952 after an outbreak on the Makonde Plateau of Tanganyika Territory (*4*), currently part of Tanzania. The word *chikungunya* is from a local dialect that translates to “that which bends up” in reference to the stooped posture often seen in patients with severe arthralgia (*5*). Human infections with chikungunya virus are associated with sudden onset of symptoms including headache, fever, rash, and muscle and joint pain (*5*). Before the outbreak in the Indian Ocean islands, deaths had not been associated with infections. However, Réunion Island reported >200 deaths during the 2006 chikungunya epidemic (January–April 2006) (*6*), and India conservatively estimated 1,194 deaths since the virus reemerged in December 2005 (*7*). To determine whether a similar situation occurred in Mauritius, we compared expected number of deaths with observed number of deaths and estimated number of chikungunya cases from January through December 2006.

Crude death rates (CDRs) and number of deaths were obtained from the Annual Digest of Statistics 2005 and 2006 (*8*). We calculated expected number of deaths for each month in 2006 by multiplying mean CDR (per 1,000 midyear population) of each month for the previous 10 years (1996–2005) by midyear population for 2006. Estimated number of cases of chikungunya fever in 2006 for the Island of Mauritius was obtained from the Chikungunya Unit of the Ministry of Health and Quality of Life and includes both suspected and confirmed cases. A case was considered suspected if the patient had >2 of the following 3 symptoms: fever, rash, and joint pain. Cases were confirmed by cell culture.

CDRs for the Republic of Mauritius ranged from 6.7 to 7.0 during 1996–2005 (mean 6.8) but increased to 7.3 in 2006, which represented a 4.3% (95% confidence interval [CI] 2.2%–8.5%) increase from 2005 (CDR 7.0). According to the 2006 midyear Population and Vital Statistics Report for the Republic of Mauritius (*8*), the number of deaths from January through June 2006 increased by 16.7% (95% CI 11.0%–23.5%) over the number of deaths in 2005, and 19.8% (95% CI 13.5%–26.9%) more deaths occurred among elderly persons (age >60 years). These numbers include all potential causes of deaths. However, increased CDRs occurred only during the months of the chikungunya fever epidemic on Rodgrigues and Mauritius Islands.

CDRs on the Island of Mauritius in March, April, and May 2006 were significantly greater than expected (p<0.01 for all 3 months); this corresponds to 743 excess deaths for these months (Figure). The chikungunya fever epidemic in Mauritius began ≈1 month before the first month of reported excess deaths; 4,871 cases of chikungunya fever cases were reported in February, 5,084 in March, and 2,305 in April. Rodrigues Island showed a similar trend, with an increase in the number of observed deaths in April, May, and June (p<0.01 for May). The first confirmed case of chikungunya fever on Rodrigues Island was reported in February, followed by 56 cases in March, 393 in April, and >80 in May (*9*).

**Figure:**
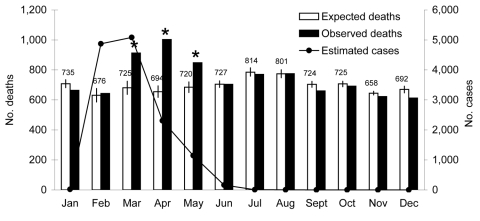
Expected and observed numbers of deaths and estimated number of chikungunya cases on Mauritius Island. Asterisks indicate a statistically significant (p<0.01) difference between expected and observed number of deaths for the specified months. Vertical lines show 95% confidence intervals; upper confidence limits are shown numerically.

The increase in CDRs reported for the Republic of Mauritius during the chikungunya epidemic is similar to the findings reported for the neighboring island of Réunion (*10*). Excess deaths in Réunion and the Republic of Mauritius coincided with the epidemic curve of the chikungunya fever outbreak, which suggested an association between these 2 factors. No other events that may have negatively affected the health of persons living in the Republic of Mauritius in 2006 were reported. However, because information on cause of death is unavailable, studies are needed to confirm that the chikungunya fever outbreak contributed to increased CDRs in 2006.
